# Amelioration of Prenatal Lead-Induced Learning and Memory Impairments by Methanolic Extract of Zataria Multiflora in Male Rats

**DOI:** 10.32598/bcn.10.2.1104.1

**Published:** 2019-03-01

**Authors:** Farahnaz Taheri, Gholamreza Sepehri, Vahid Sheibani, Fariba Sharififar

**Affiliations:** 1. Neuroscience Research Center, Institute of Neuropharmacology, Kerman University of Medical Sciences, Kerman, Iran.; 2. Department of Physiology and Pharmacology, Faculty of Medicine, Kerman University of Medical Sciences, Kerman, Iran.; 3. Department of Pharmacognosy, Faculty of Pharmacy, Kerman University of Medical Sciences, Kerman, Iran.

**Keywords:** Lead, Prenatal exposure, Zataria Multiflora, Spatial learning and memory, Rats

## Abstract

**Introduction:**

The current study aimed at evaluating the effects of Zataria Multiflora (ZM) on learning and memory of adult male offspring rats with prenatal lead-exposure.

**Methods:**

Pregnant rats in the case group received tap water containing 0.2% lead acetate throughout the gestation period. Control rats had free access to lead-free tap water. Two male offspring (two-month-old, weighing 180–200 g) from each mother were randomly selected and treated with either Z. Multiflora (50, 200, 400, and 800 mg/kg/Intraperitoneally (I.P)/20 day) or saline. Spatial memory of the control, saline, and ZM-treated rats was evaluated by a training trial and probe test using Morris water maze (6–8 rat/group).

**Results:**

The obtained results showed memory deficits including increased escape latency, and a greater traveled distance, as well as decrements in the frequency of crossings into target quadrants in prenatally lead-exposed male offspring compared with the controls. ZM treatment (200 mg/kg/i.p) ameliorated the memory deficits in male offspring by increasing the time spent and traveled distance in the trigger zone (P<0.01 vs. saline).There was no significant difference in swimming speed between the groups.

**Conclusion:**

The results showed memory deficits in prenatally lead-exposed male offspring. ZM treatment (especially 200 mg/kg) had beneficial effects on cognitive behavior and was indicated as the improvement of lead-induced memory deficits in prenatally lead-exposed male rats. The exact mechanism(s) is not determined yet, but it could be mediated through the anticholinesterase and antioxidant effects and also alterations in Central Nervous System (CNS) and neurotransmission in the central nervous system.

## Highlights

- There were memory deficits in antenatally lead-exposed male offspring rats.
- Prenatal lead-exposure caused spatial memory deficits in Morris water maze test in male offspring rats.
- Zataria Multiflora (ZM) ameliorated memory deficits in antenatally lead-exposed male offspring rats.
- ZM beneficial effects may be mediated through its anticholinesterase and antioxidant effects.

## Plain Language Summary

Lead is a heavy metal in the environment and lead exposure is a major public health problem, especially in children. Exposure to lead can occur by contaminated air, water, dust, food, or consumer products. The brain is the most sensitive organ to lead poisoning and exposure in early life is associated with memory and cognitive function impairments in children, adolescents, and young adults. Removal of the lead source is the main prevention of lead-induced impairments. Herbal plants have been used to improve memory and cognitive function in many countries. Zataria Multiflora (ZM), commonly known as “Avishan Shirazi” in Iran, is known for its beneficial effects on mental abilities. In this study, we evaluated the effects of “Avishan Shirazi” on the spatial memory of male offspring rats born from mothers exposed to lead-contaminated drinking water during pregnancy. Our results showed that lead exposure during pregnancy significantly impaired learning in adult offspring. Lead-induced impairments in adult offspring were partially reversed by “Avishan Shirazi” treatment. It showed beneficial effects on the memory of prenatally lead-exposed adult male rats. However, its exact mechanism(s) is not determined yet and needs further research.

## 1. Introduction

Lead exposure remains as one of the most important problems in terms of relevance of exposure and a major public health risk, particularly in the developing countries (Hu, Shih, Rothenberg, & Schwartz, 2007; Flora, Gupta, & Tiwari, 2012). Lead exposure in early life is among the major causes of the persistent decrements in intelligence documented in children, adolescents, and young adults, as well as the development of neurodegenerative disease in life (Glass et al,. 2009).

Prenatal lead exposure is associated with adverse effects on neurodevelopment and the effect is most pronounced during the first trimester of pregnancy in which the maternal plasma of lead level in pregnant women (Hu et al., 2006) is needed. Also, accumulated exposure to lead is associated with cognitive decline in the elderly (Glass et al,. 2009).

It is reported that exposure to lead during pregnancy and lactation induces neurobehavioral effects, hyperactivity, decreased exploratory behavior, and deficits in learning and memory in adult offspring (Gardella, 2001). Lead exposure during childhood could impair both physical development and increase in bone resorption (Yang et al., 2013). Previous studies showed that very low-level of prenatal lead exposure was associated with a significant impairment in cognitive function in boys at 36 months (Jedrychowski et al., 2009). Also, blood lead concentrations lower than 5 μg/dL are associated with deficits in cognitive and academic skills in children aged 6–16 years (Lanphear et al., 2005).

The disturbances in learning ability, adaptive responses, and other aspects of behavior following lead exposure may be related to significant changes in some Central Nervous System (CNS) neurotransmitters such as glutamate, dopamine, and acetylcholine (NourEddine, Miloud, & Abdelkader, 2005; Sidhu & Nehru, 2003). Lead exposure causes significant decrease in the activity of the acetylcholinesterase and other neurotransmitters in rat brain (Sidhu & Nehru, 2003).

Management of lead-induced biochemical and behavioral changes during prenatal lead exposure focuses on removal of the lead source. Sometimes the removal of maternal lead sources is needed by chelation therapy. Recognition and removal of lead sources during the prenatal period can prevent maternal and neonatal morbidity (Gardella, 2001).

The pathogenesis of lead poisoning may be mediated through decrease in the activity of the acetylcholine (Sidhu & Nehru, 2003) and disturbance between the delicate pro-oxidant/antioxidant balance that exists within mammalian cells in brain. Production of Reactive Oxygen Species (ROS) is increased after lead exposure in in vitro studies as well as in vivo ones and alteration of anti-oxidant defense systems in animals and occupationally exposed workers (Hsu & Guo, 2002).Therefore, exogenous supplementation of antioxidant molecules may be among the strategies to decrease lead-induced toxicities (Sidhu & Nehru, 2003).

Today, herbal plants are an important part of traditional medicine in many countries and they are used to improve memory and cognitive function in traditional medicine. Various studies show that plants with high antioxidant and acetylcholinesterase effects could be effective in the treatment of memory disorders. One of these plants can be named Zataria Multiflora (ZM) (Sharififar, Mirtajadini, Azampour, & Zamani, 2012).

Zataria Multiflora Boiss (ZM), commonly known as Avishan Shirazi in Iran, is a member of Laminaceae family that grows only in Iran, Pakistan, and Afghanistan and is used in Iranian traditional medicine for its beneficial effects on mental abilities (Majlessi, Choopani, Kamalinejad, & Azizi, 2012). Experimental studies showed that amyloid β Induced cognitive deficits were reversed by ZM essential (Majlessi et al., 2012).The beneficial effects of ZM seem to e contribute to its antioxidant, anti-inflammatory and anticholinesterase activities of ZM or its main constituents (Majlessi et al., 2012; Sharififar et al., 2012; Sharififar, Moshafi, Mansouri, Khodashenas, & Khoshnoodi, 2007).

Thymol, a phenolic compound of oxygenated monoterpens and carvacrol are the main constituents of the dry plant while the main constituents of the fresh plant include thymol, carvacrol, p-cymene, linalool, and gamma-terpinene (Saei-Dehkordi, Tajik, Moradi, & Khalighi-Sigaroodi, 2010; Zomorodian et al., 2011). It is reported that ZM extracts had significant and dose-dependent antinociceptive activity in mice. Also, ZM had remarkable activity against acute inflammation induced by acetic acid in mice and a dose-dependent and significant antinociceptive activity in hot-plate and writhing tests (Hosseinzadeh, Ramezani, & Salmani, 2000; Ramezani, Hosseinzadeh, & Samizadeh, 2004).

Prenatal lead exposure as well as environmental lead exposure in childhood is associated with a reduced intellectual development, significant decrease in brain volume, cognition deficits, executive functions, social behaviors, and motor abilities in adults (Schnaas et al., 2006; Lanphear et al., 2005); therefore, the prevention of lead-induced biochemical and behavioral changes during prenatal lead exposure is a worldwide strategy, and there is an increasing trend to use herbal medicine to prevent or treat cognitive impairments. Since ZM is used in Iranian traditional medicine for its beneficial effects on mental abilities, the current study aimed at evaluating the effects of ZM on prenatal lead-induced learning and memory deficits in male offspring rats.

## 2. Methods

### 2.1. Animals

All experimental procedures approved by the Ethics Committee of Kerman Neuroscience Research Center (Ethics Code: KNRC-92-57). Female Wistar rats (3–4 months old, weighing 250–300 g) were used for the current study. Animals were caged in groups of six with ad libitum access to food and water for two weeks before mating. They were housed under controlled temperature (23±1ºC) and 12:12-hour light/dark cycle (7:00–19:00 light).Two females were paired in a cage with a male rat in the late afternoon.

The next morning the female rats were examined for the presence of vaginal plugs. The day in which vaginal plugs was observed, was designated as the Gestation Day of 0 (GD 0).Then, pregnant rats were randomly divided into two groups: Control group, which had free access to tap water and Gestation group (G) in which tap water was replaced with a solution containing 0.2% lead acetate from the first day of gestation and continued until parturition, when tap water was restarted (Rossouw et al., 1987). The number of litters was adjusted to eight for each dam.

The offspring were kept with their mothers and then two male offspring from each mother (two months old, weighing 180–200 g) were randomly selected to evaluate ZM effects on their learning and memory behavior (6–8 rat/group).

### 2.2. Preparation of Zataria Multiflora extract

Z. Multiflora Boiss aerial parts were purchased from a local market in Kerman, Iran, in June 2014 during the spring season. The plant was identified and confirmed by the Pharmacognosy Department of School of Pharmacy (Kerman, Iran) as Z. Multiflora Boiss (Laminaceae family) and air-dried and powdered in a grinder. Methanolic extract of Z. Multiflora was prepared by socking 250 g of the powdered plant in 1000 mL of 85% methanol using percolation method for 48 hours. The solution thereafter was filtered and the filtrate was evaporated in an oven at 40ºC. Solvent removal was conducted under vacuum and afforded a semisolid mass with a yield of 8.6%. The extract was dissolved in distilled water to prepare a solution containing 1000 mg/mL concentration from dry weight of Z. Multiflora.

### 2.3. Experimental groups

Male rat offspring were randomly divided into six groups: Control group: Male offspring of control groups with no prenatal exposure to lead, which received intraperitoneal injection of normal saline for 20 days. SAL group: Male offspring with prenatal exposure to lead, which received intraperitoneal injection of saline for 20 days. Treatment groups: Male offspring with prenatal exposure to lead, which received intraperitoneal injection of methanolic extract of Z. Multiflora (50, 200, 400, and 800 mg/kg) for 20 days.

### 2.4. Morris water maze apparatus and procedures

After the last treatment on day 20, male offspring were subjected to behavioral testing for spatial learning and memory using Morris Water Maze (MWM). In most studies MWM is used to evaluate neural mechanisms of spatial learning and in animal models 22. MWM apparatus is a circular black and transparent pool painted with nontoxic materials (160 cm diameter, 80 cm height and 40 cm depth, filled with 22±1°C water).

The maze was divided geographically into four equal quadrants and held release points were designed at each quadrant as N, E, S, and W. A square hidden black platform (10 cm diameter) was submerged beneath (1.5 cm) the water surface in the middle of the target quadrant in the pool. Visual cues were placed around the pool. The animal motion was recorded and sent to the computer by a camera mounted above the center of the maze. The swimming speed and time latency to reach the platform and also the length of swimming pathway were recorded semi-automatically by a commercial software (Noldus, Netherlands; XT V. 6).

### 2.5. Spatial learning measurements procedure

Each animal was handled daily for three days prior to the initiation of the experiments. Then the rats were habituated to the water maze for 60 seconds without a platform. Each rat performed a trial test daily for four consecutive days. In each trial, the rats were placed randomly at the middle of the circular edge in a randomly selected quadrant in MWM apparatus and released facing the side wall at one of the four positions (the boundaries of the four quadrants, labeled N, S, E, and W).

On each trial, the rat was allowed to swim until it found and remained on the platform for 20 seconds. If animal did not find the platform after 60 seconds the experimenter would assist the rat to find the platform and allowed to stay on the platform for 20 seconds. Then the rat was removed from the pool, dried with a towel and located in its holding cage. The next trial was performed after 20–30 seconds of animal rest. Parameters such as latency and the traveled distance to find the platform were recorded in each trial.

### 2.6. Probe test evaluation

Probe test was performed to evaluate spatial memory retention on the 5^th^ day (24 hours after the last trial). The experimenter conducted a probe trial in which the escape platform was removed from the pool and each rat was placed into the pool from the opposite quadrant and allowed to swim freely for 60 seconds. Usually, the welltrained rats spent most of the swimming time in the target quadrant of the pool across the former location of the platform. The time swum in target quadrant, swimming speed, and the number of times rat crossed the platform area in the probe phase were recorded.

A visible platform test was performed to evaluate any possible sensory and motor coordination deficits or motivation abnormality of male offspring. In this test, the ability of animals to escape to a visible platform was evaluated (the platform was raised 2 cm above the water level and was visible with aluminum foil) (Hajaliet et al., 2012).

### 2.7. Plasma lead measurement

In another set of experiments, lead levels were evaluated in separate groups of pregnant rats (seven rats/group) randomly divided into two groups: Control group with free access to tap water, and Gestation (G) in which tap water was replaced with a solution containing 0.2% lead acetate from the first day of gestation and continued until parturition (Rossouw, Offermeier, & Van, 1987). Blood samples from both control and lead-exposed rats were collected from orbital sinus after parturition and serum concentration of lead was measured by atomic absorption spectrophotometry (Parasuraman, Raveendran, & Kesavan, 2010).

### 2.8. Statistical analysis

The data were presented as Mean±SEM of the seven rats in each group. T-test was used to compare the mean differences between the two groups and two-way Analysis of Variance (ANOVA) test was used to compare the mean differences between the groups. Tukey post hoc multiple comparison test was performed to assess differences between the experimental groups. P<0.05 was considered as the level of significance.

## 3. Results

### 3.1. Serum lead level in pregnant rats

The current study results showed significant difference in the serum lead concentration in the control and leadexposed mother rats (235.76 μg/L) by atomic absorption spectrophotometry (Table 1).

**Table 1.** Serum lead level in pregnant rats

**Group**	**Mean Level of Blood Lead (μg/L)**
Control	0
Lead	235.76

Pregnant rats received a solution containing 0.2% lead acetate from the 1^st^ day of gestation and continued until parturition. Control group had free access to tap water.

Control, control

### 3.2. Zataria Multiflora analysis by GC/MS

Authors previously reported that thymol (37.59%), carvacrol (33.65%); paracymene (7.72%), γ-terpinene (3.88%). and β-caryophyllene (2.06%) were the main components of Z. Multiflora comprising 84.9% of the oil.13.

### 3.3. Spatial learning in prenatally lead-exposed male rats

**Figure 1.**
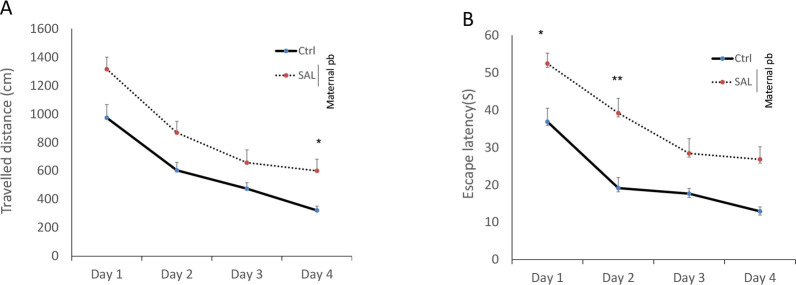
The effects of lead on memory of antenatally lead exposed male offspring rats during 4 consecutive days The effects of lead on traveled distance (A); the average escape latency (B) during spatial learning to find the hidden platform in the Morris water maze in antenatally lead-exposed adult male offspring. Adult male offspring rats, Control, and SAL groups received saline. Data are expressed as Mean±SEM (7 rats/group). Ctrl: Control; SAL: Saline; Maternal pb, lead exposure in prenatal period; ^*^P<0.05; ^**^P<0.01 vs. Control

**Figure 2.**
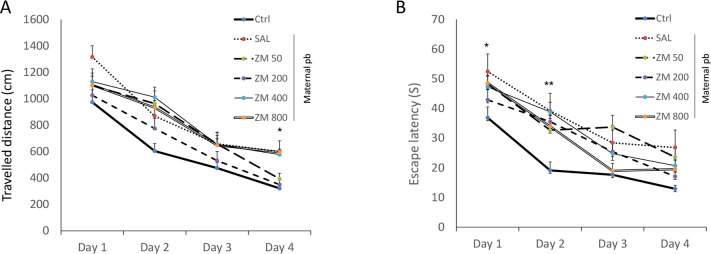
The effects of ZM on memory of antenatally lead exposed male offspring rats during 4 consecutive days The effects of Zataria Multiflora on traveled distance (A); and the average escape latency (B) during spatial learning to find the hidden platform in the MWM in antenatally lead-exposed adult male offspring. Adult male rats offspring received Z. Multiflora (50, 200, 400, and 800 mg/kg/intraperitoneally (i.p.)/20 d). Control and SAL groups received saline. Data are expressed as Mean±SEM (7 rats/group).

The current study results showed training impairment in prenatally lead-exposed male rats (SAL group) vs. control rats. Analysis of traveled distance at first, second, third, and fourth days of training (four trials per day), using repeated measures two-way ANOVA revealed a significant training progress through the four days of experiment [F(5, 42)=93.817, P<0.001] (Figure 1 and 2).

During the acquisition phase, as shown in Figure 1, the data showed a significant difference in the traveled distance in SAL animals vs. Control on the 4th day; [F(5, 42)=2.497, P<0.05] (Figure. 1 A). Also, the SAL animals (prenatally lead-exposed male rats) needed more time to find hidden platform than the Control rats (on the first day; [F(5, 42)=2.261, P<0.05], second day; [F(5, 42)=3.304, P<0.01]) (Figure 1 B).

### 3.4. The effects of ZM (50, 200, 400, and 800 mg/kg) on the average escape latency to find the hidden platform and traveled distance in antenatally lead-exposed male offspring (spatial learning)

As shown in Figure 2, during the acquisition phase, there was no significant difference in the traveled distance and escape latencies between ZM treated rats with those of the Control and SAL.

### 3.5. The effects of ZM on traveled distance, the time spent, and crossings to target quadrants in antenatally lead-exposed male offspring during the probe test (memory retention)

**Figure 3.**
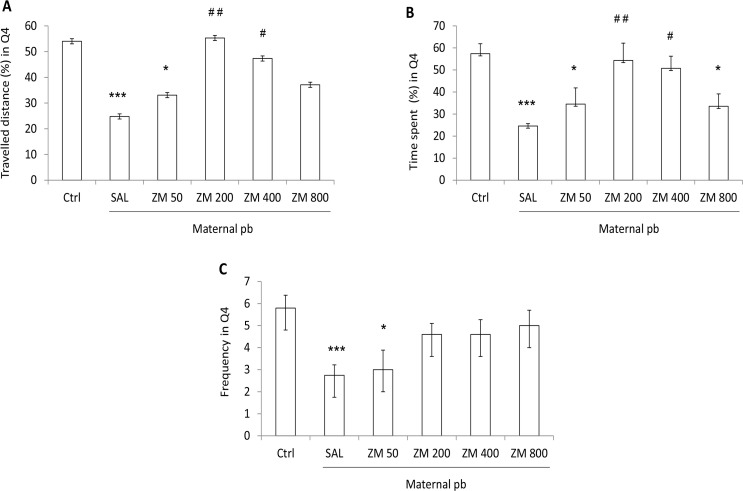
The effects of ZM on memory of antenatally lead exposed male offspring rats during the probe test (memory retention) The effects of Zataria Multiflora on the traveled distance (A); the time spent (B); and the crossings (C) in trigger zone in antenatally lead-exposed adult male offspring during the probe test (memory retention). Adult male offspring received Z. Multiflora (50, 200, 400, and 800 mg/kg/i.p./20 d). Control and SAL groups received saline. Data are expressed as Mean±SEM (7 rats/group). Ctrl: control; SAL: saline; maternal pb, lead exposure in prenatal; ^*^P<0.05; ^***^P<0.001 vs. Control group; ^#^P<0.05; ^##^ P<0.01 vs. SAL group

Data showed that the traveled distance in trigger zone in SAL and ZM 50 was significantly lower than that of the Control (P<0.001, P<0.05, respectively) (Figure 3 A). ZM 200 and ZM 400 significantly increased the traveled distance in trigger zone vs. SAL (P<0.01, P<0.05, respectively) (Figure 3 A).The time spent in trigger zone in SAL, ZM 50, and ZM 800 was significantly lower than those of the Control rats (P<0.001, P<0.05, P<0.05 in SAL; ZM 50 and ZM 800 vs. Control, respectively) (Figure 3 B). Treatment with ZM 200 and ZM 400 resulted in a sinificant increase in the time spent in trigger zone compared to that of the SAL rats (P<0.01, P<0.05, respectively) (Figure 3 B). There was a significant difference in crossings to target quadrants among SAL (P<0.001) and ZM 50 (P<0.05) vs. Control rats (Figure 3 C). The swimming speed in the target quadrant showed no significant difference among Control, SAL, and ZM treated offspring.

### 3.6. Escape latency to find visible platform

The current study results showed no significant difference in the time spent to find the visible platform in Control, SAL, and ZM treated male offspring (Table 2).

**Table 2.** The time spent to find the visible platform in antenatally lead-exposed adult male rat offspring

**Group Escape Latency (S)**	**Mean±SEM**
Control	31.1±4.16
SAL	37±6.3
ZM 50	43.7±4.03
ZM 200	52.1±4.9
ZM 400	43.4±7.1
ZM 800	33.6±4.3

Comparisons of escape latency to escape on to the visible platform in MWM in antenatally lead-exposed adult male offspring, using one-way ANOVA (the differences were not significant). Adult male offspring received Z. Multiflora (50, 200, 400, and 800 mg/kg/i.p./20 d). Control and SAL groups received saline. Data are expressed as Mean±SEM of 8 rats/group.

SAL: saline; ZM: Zataria Multiflora; maternal pb, lead exposure in prenatal

## 4. Discussion

Lead exposure is one of the most important public health risks during the first trimester of pregnancy in most countries associated with a significant impairment in cognitive function both in childhood and adolescence period. Therefore, prevention of lead-induced behavioral changes during prenatal lead-exposure is the best possible strategy. In the current study, the effects of methanolic extract of ZM on lead-induced deficits in cognitive behavior of prenatally lead exposed male offspring were evaluated by MWM. The data revealed elevated serum lead concentration during the gestation period (235.76 μg/L).

The current study results indicated that lead-exposure during gestation period resulted in long lasting deficits in spatial reference learning in adult male offspring. Escape latency to find the hidden platform, as an indication of learning index, significantly increased in prenatally lead-exposed male offspring. Also, the results of the probe test showed a significant increase in escape latency, travelled distance, as well as decrements in the crossing frequencies to target quadrants in the prenatally lead exposed male offspring. These deficits in cognitive behavior can be attributed to spatial performance. However, there was no significant difference in the swimming speed and ability of rats to find the visible platform, which showed that prenatal lead exposure did not affect the locomotion or sensorimotor coordination.

The current study data were in complete agreement with those of previous reports and lead-induced cognitive behavior impairments were reported previously both in human and animal studies (Flora et al., 2012; Glass et al,. 2009; Hu et al., 2006; Zhi-wei, Ru-Lai, Guijuan, D., & Zhengyan, 2005). The cellular, intracellular, and molecular mechanisms of lead neurotoxicity are numerous and the possible mechanisms by which lead exposure causes impaired learning and memorizing abilities may be mediated through different pathways. Lead exposure during pregnancy results in high lead blood level in umbilical cord blood and fetus blood level, which cause neurobehavioral impairments in infants and children by affecting the anticholinesterase activity (Sharififar et al., 2007; Majlessi et al., 2012; Reddy, Devi, & Chetty, 2007).

Lead exposure results in a significant decrease in the intensity of anticholinesterase staining in the dentate gyrus, CA2, and CA3 areas of hippocampus, as well as the different cell layers of cortex and cerebellum (Reddy, Devi, & Chetty, 2007). Alteration in the CNS neurotransmitters such as dopamine, glutamate, serotonin, and norepinephrine are also involved in lead-induced behavioral changes (NourEddine et ai., 2005; Sidhu & Nehru, 2003; Reddyet al., 2007). Also lead-induced toxicity may be mediated through the production of ROS confirmed in both in vitro and in vivo studies in animals and occupationally exposed workers (Hsu & Guo, 2002). Nitric Oxide Synthase (NOS) activity in the hippocampus, the cerebral cortex, and the cerebellum of rats were inhibited by low-level lead exposure (drinking water containing 0.025%, 0.05% and 0.075% lead acetate/28 d)25. Lead toxicity could result in oxidative stress, DNA damage, and apoptosis (Yedjou, Milner, Howard, & Tchounwou, 2010).

The lead-induced cognitive deficits were reversed by administration of ZM essential oil. The beneficial effects of ZM (200 mg/kg) on spatial memory was characterized by the increased travelled distance and time spent in quadrant zone as well as an increase in crossings to target quadrants in the prenatal lead-exposed rats.

The mechanism(s) by which ZM ameliorate the lead-induced cognitive impairments in antenatally lead-exposed rats is not precisely determined; however, the current study GC/MS analysis of methanolic extract of ZM essential oil showed that thymol (37.59%) and carvacrol (33.65%) were the main constituents of the dry plant of ZM essential oil, therefore, the beneficial effects of ZM on cognitive behavior of rats could be mainly mediated by these compounds (Sharififar et al., 2007; Majlessi et al., 2011). Others also reported that the carvacrol and thymol as the main constituents of ZM essential oil (Azizi, Ebrahimi, Saadatfar, Kamalinejad, & Majlessi, 2012; Kavoosi & Rowshan, 2013) reported that thymol and carvacrol alleviate the cognitive deficits induced by Amyloid β (Aβ) or scopolamine in the rat models of dementia. The beneficial effects of Z.

Multiflora on rat models of dementia may be mediated through its anticholinesterase, antioxidant, and anti-inflammatory activities (Azizi et al., 2012). Although both carvacrol and thymol possess anticholinesterse activity, however, anticholinesterse inhibitory effect exerted by carvacrol is reported to be 10 times stronger than that of its isomer thymol (Jukic et al., 2007). Amelioration of cognitive deficits by ZM is reported by other investigators (Majlessi et al., 2011; Sharififar et al., 2012). Gelatin films prepared from gelatin solutions (10% w/v) containing ZM essential oil exhibited excellent antioxidant and antimicrobial properties (Kavoosi, Rahmatollahi, Dadfar, & Purfard, 2014).

Kavoosi et al. (2012) reported that ZM essential oil, thymol and carvacrol, significantly reduced Nitric Oxide (NO) and Hydrogen peroxide (H_2_O_2_) production in Lipo Poly Saccharide (LPS) stimulated macrophages and thus could be potentially used in the therapy of lead-induced oxidative damage mechanism of cognitive deficits (Hassanshahi, Roghani, & Raoufi, 2014; Kavoosi et al., 2012) Carvacrol, the main constituent of ZM, antioxidant, and NO scavenging, and malondialdehyde scavenging activities may be involved in the reversing of lead-induced cognitive impairments in rats (Kavoosi et al., 2012). Zotti et al. (2013) showed that carvacrol, increased dopamine and serotonin levels in the prefrontal cortex and hippocampus in rats and thus can clearly influence behavioral outcomes through modulation of neurotransmitters.

In summary, the current study showed that lead exposure during pregnancy caused impaired memory of male rat offspring in MWM test. Administration of Z. Multiflora (200 mg/kg) improved lead-induced memory deficits in prenatally exposed male rats. The exact mechanism(s) underlying the beneficial effects of ZM on lead-induced memory impairment is not determined yet, but it could be mediated through the anticholinesterase activity, antioxidant effects, nitric oxide scavenging and malondialdehyde scavenging activities, and alterations in CNS neurotransmission such as dopamine, glutamate, serotonin and norepinephrine in the Central Nervous System by carvacrol/thymol, the main constituents of ZM. Further studies are needed to elucidate the underlying mechanism(s).

## Ethical Considerations

### Compliance with ethical guidelines

All experimental procedures were performed in accordance with the guidelines provided by the experimental animal laboratory and approved by the Ethics Committee of Kerman Neuroscience Research Center (Ethics Code: KNRC-92-57).
